# Assessing *Alternaria* Species and Related Mycotoxin Contamination in Wheat in Algeria: A Food Safety Risk

**DOI:** 10.3390/toxins17060309

**Published:** 2025-06-18

**Authors:** Meriem Barkahoum Daichi, Mario Masiello, Miriam Haidukowski, Annalisa De Girolamo, Antonio Moretti, Amor Bencheikh, Noureddine Rouag, Stefania Somma

**Affiliations:** 1 Laboratory of Applied Microbiology, Department of Agronomy, Faculty FSNV, University Ferhat Abbas-Setif 1, Sètif 19137, Algeria; marydaichi@gmail.com (M.B.D.); n.rouag@univ-setif.dz (N.R.); 2 Institute of Sciences of Food Production, National Research Council (CNR-ISPA), via Amendola 122/O, 70126 Bari, Italy; mario.masiello@cnr.it (M.M.); edithmiriam.haidukowski@cnr.it (M.H.); annalisa.degirolamo@cnr.it (A.D.G.); antonio.moretti@cnr.it (A.M.); 3 Department of Microbiology, Faculty FSNV, University Ferhat Abbas-Setif 1, Sètif 19137, Algeria; benchomar@univ-setif.dz

**Keywords:** *Alternaria eureka*, *A. tellustris*, black point, arid climatic regions, alternariol, alternariol monomethyl ether, tenuazonic acid

## Abstract

*Alternaria* species are important fungal pathogens occurring worldwide in wheat, causing both productive and qualitative losses, and posing a toxicological risk to human health due to the production of their mycotoxins in kernels. This study aimed to investigate the occurrence of *Alternaria* species and their mycotoxins in 48 wheat grain samples collected from the northeast to the southeast of Algeria. Seventy-two representative *Alternaria* strains were molecularly analyzed using a multi-locus sequence approach and evaluated for their capability to produce mycotoxins under in vitro conditions. *Alternaria alternata*, representing 42% of the strains, was the dominant species, followed to a lesser extent by species included in the *Infectoriae* section (26%). In addition, three species not previously reported in Algerian wheat, *A. eureka*, *A. consortialis* and *A. tellustris*, were identified, accounting for 5% of the total strains. Mycotoxin analyses showed high contamination of grains with alternariol monomethyl ether, alternariol and tenuazonic acid, occurring in 75, 69 and 35% of the samples, respectively. Moreover, 41 out of 48 samples showed the co-occurrence of multiple *Alternaria* mycotoxins. This study provides, for the first, time a clear picture of the occurrence and the distribution of *Alternaria* species on wheat in Algeria. Finally, the extensive monitoring activities carried out revealed the great biodiversity of *Alternaria* species able to colonize wheat grains. Moreover, findings on mycotoxin contamination raise concerns about the significant mycotoxigenic risk in Algerian wheat, emphasizing the need for strict monitoring and regulatory measures on *Alternaria* mycotoxins in food and feed.

## 1. Introduction

Durum wheat, one of the earliest domesticated plants worldwide, was introduced in the Mediterranean Basin about 2000 years ago, representing a staple food for the human diet [1]. In Algeria, wheat production remains below average, estimated at approximately 2.5 million tonnes in 2023, reflecting a 17% decrease from the previous year [2]. The yield and the quality of wheat grains can be influenced by agronomic practices and several abiotic and biotic stresses, including microorganisms able to colonize wheat during the whole crop season as well as during storage. Among these, mycotoxigenic fungi are of particular concern for both their phytopathological issue and their capability to produce mycotoxins that accumulate in the grains. Multiple fungal species, mainly belonging to *Fusarium* and *Alternaria* genera, can co-occur on wheat grains at harvest, persisting through storage and leading under suitable conditions to the accumulation of a broad range of mycotoxins in the kernels [3].

Black Point, also known as kernel smudge, is a severe disease of wheat, characterized by dark discoloration at the embryo end of wheat kernels and detected in all cereal-cultivated areas [4]. Black Point development in wheat is influenced by agricultural practices and environmental conditions, with excessive irrigation or rainfall after flowering increasing the incidence and its severity in susceptible cultivars [5]. Although *Alternaria* species are the main causal agents of Black Point of wheat, other fungi such as *Cladosporium*, *Bipolaris* and *Fusarium* have also been detected from diseased kernels [4]. *Alternaria* species are common saprophytes or pathogens widely spread in atmosphere, soil and on several plants, including cereals, due to their great ability to adapt to environmental factors [6]. The most frequently detected *Alternaria* species occurring on wheat kernels are included in the *Alternaria* and *Infectoriae* sections, with *A. alternata* isolated with higher frequency worldwide [7,8,9]. Moreover, *A. tricitina*, a member of the *Infectoriae* section, the most important causal agent of wheat leaf blight in Argentina, India and China, is considered among the main species involved in Black Point [10]. The genus *Alternaria* includes more than 250 species, which have mostly been identified based on morphological traits, including shape, color, size, septation and sporulation [11]. However, due to the great plasticity of *Alternaria* species influenced by growing conditions, morphological identification is extremely laborious and time consuming and could lead to a misidentification [12,13]. Recent advances in molecular approaches, combined with morphological traits, genetic markers and mycotoxin profiles, have led to a more in-depth revision of the taxonomy of the genus *Alternaria*. In particular, the use of multi-locus phylogenetic analyses has enabled the identification of new species within the *Alternaria* section [14], thus allowing for a deeper understanding of the genus, which already is known to comprise a substantial number of species distinguished by different morphological traits, host plants and molecular data. The *Alternaria* section includes around 60 morphospecies, although polyphasic taxonomic approaches have narrowed this classification to 11 accepted species and one species complex (*A. arborescens*), while 35 morphospecies were synonymized as *A. alternata* [15].

*Alternaria* species can produce more than 70 secondary metabolites, including mycotoxins, with toxic properties under certain conditions of temperature and humidity [16]. The contamination of crops by *Alternaria* and the subsequent accumulation of their toxic metabolites in food and feed have been thoroughly investigated [4,8,9,17,18]. These metabolites belong to five different structural groups, based on their chemical structure: (1) dibenzopyrone derivatives, including alternariol (AOH), alternariol monomethyl ether (AME) and altenuene (ALT); (2) perylene derivative including altertoxins (ATX-I, ATX-II and ATX II); (3) tetramic acid derivative, like tenuazonic acid (TeA); (4) cyclic tetrapeptide such as tentoxin (TEN); (5) AAL toxins, produced by *A. alternata* f. sp. *lycopersici* [19]. Among these compounds, TeA, AOH, AME, ALT and ATX-I are the main *Alternaria* mycotoxins frequently occurring in food commodities [20].

Studies on mutagenicity and carcinogenicity of AME and AOH established that these mycotoxins can play an important role in the etiology of human esophageal cancer [21]. In addition, AOH has been reported to possess cytotoxic, genotoxic and mutagenic properties in vitro [22] and to increase the production of reactive oxygen species, leading to oxidative stress and subsequent cell damage [21].

Despite the wide occurrence of *Alternaria* species and their mycotoxins in crops and food commodities, currently there are no legal limits for the presence of *Alternaria* mycotoxins in food and feed, except for the European Union, which has published recommendations for the maximum allowable concentrations of AOH, AME and TeA in a few specific food products [23].

To date, the occurrence of *Alternaria* species and the accumulation of related mycotoxins on wheat kernels in Algeria are poorly investigated. Moreover, only a few studies deal with the distribution of fungal species in Algerian wheat [24], mainly focusing on *Fusarium* species [25,26].

This study aimed to: (a) identify the *Alternaria* species occurring on wheat in Algeria using a DNA-based approach; (b) evaluate the mycotoxin production of representative strains isolated from Algerian wheat; (c) assess the level of mycotoxin contamination in wheat samples from different Algerian regions.

## 2. Results

### 2.1. Alternaria Contamination in Durum Wheat Samples

**Figure 1:**
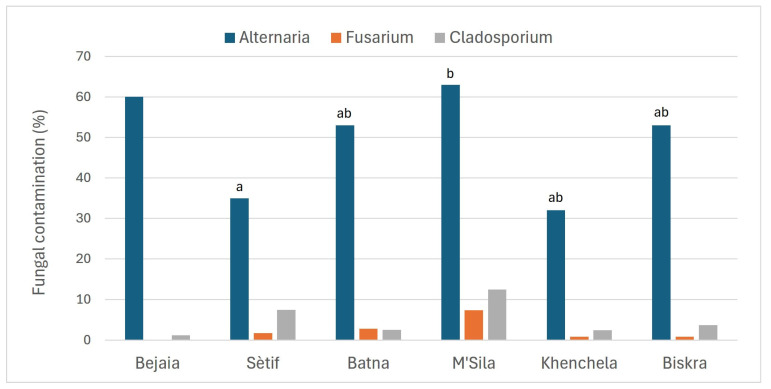
Fungal contamination by *Alternaria*, *Fusarium* and *Cladosporium* genera, expressed as a percentage, detected on wheat kernels collected in six different southeastern regions of Algeria. *Alternaria* contamination mean values with a different letter, compared using the Kruskal–Wallis test with SPSS Statistics software (v. 29.0.1.0), indicate significant differences between regions at the 0.05 level of significance.

**Figure 2:**
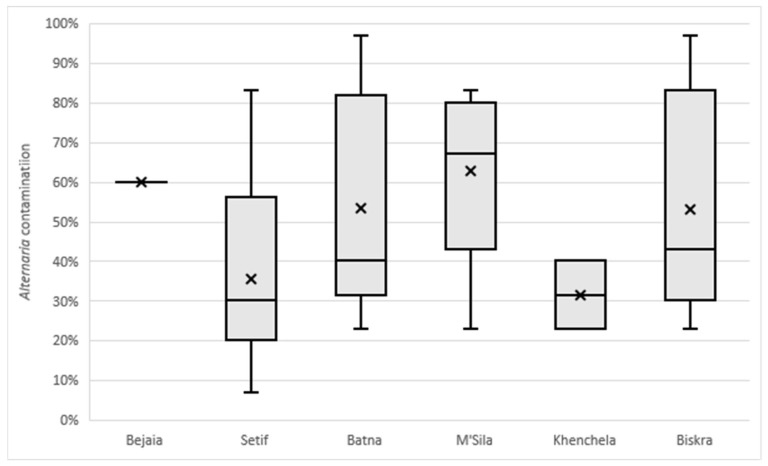
Contamination by *Alternaria* species, expressed as a percentage, detected on wheat kernels collected in six southeastern regions of Algeria. In each box plot, the limits of the whiskers show minimum and maximum values, X represents the mean value and the line inside the boxes represents the median value.

All wheat kernel samples exhibited a high level of fungal contamination, ranging from 80% to 100%. Contamination by *Alternaria*, *Fusarium* and *Cladosporium* genera was detected in all six monitored Algerian regions, with *Alternaria* representing the most prevalent one. In particular, *Fusarium* and *Cladosporium* were detected at very low levels, with mean values up to 7 and 13%, respectively, observed in the M’Sila region (Figure 1). On the contrary, *Alternaria* was detected with contamination levels ranging from 7%, in the sample V18 from the Sètif region, to 97%, in the samples V13 and V24, located in the Biskra and Batna regions, respectively (Table S1). Mean values for each region, shown in Figure 1 and Figure 2, varied from 32–35% in Khenchela and Sètif, respectively, to 53% in Batna and Biskra and reached 63% in M’Sila. Although the number of samples collected was limited (one and two samples from Bejaia and Khenchela, respectively, Table 1), the results reveal an interesting variability in *Alternaria* contamination levels across the monitored regions (Figure 2). Moreover, in each region, the samples showed very variable contamination levels (Figure 2). While preliminary, these findings highlight the need for further investigations to confirm the observed trends.

**Figure 3:**
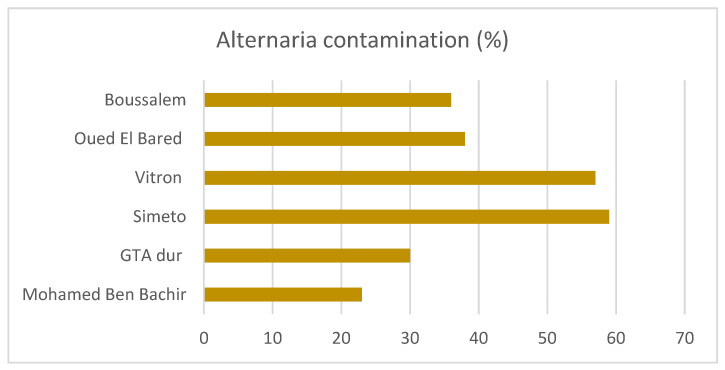
Percentage of *Alternaria* contamination in six different wheat varieties grown in Algeria.

Regarding the potential influence of wheat varieties on *Alternaria* contamination, as shown in Figure 3, the highest incidence of *Alternaria* was recorded in two varieties, i.e., Simeto, grown in the Batna region, and Vitron, grown in the M’Sila, Khenchela and Biskra regions, with mean contamination levels of 59 and 57%, respectively.

Among the 608 *Alternaria* isolates obtained from wheat kernels, 72 of them were selected as representative of the entire population based on morphological traits, such as sporulation pattern, conidia shape and size. These isolates were used for further genetic and chemical analyses.

### 2.2. Molecular and Phylogenetic Analyses of Alternaria Strains

All 72 *Alternaria* strains examined gave the expected PCR products of about 600 bp when amplified with the primers gpd1/gpd2. On the contrary, when amplified with the Alt-for/Alt-rev primer pair, 66 strains gave the expected product of 500 bp, while six out of seventy-two strains failed to amplify the fragment. Consequently, phylogenetic analysis, based on a multi-locus sequence approach, was carried out on 66 *Alternaria* strains. The aligned sequences of the *gpd* and *alt-a1* genes resulted in common fragments of approximately 470 and 400 bases, respectively. To resolve the phylogenetic relationships among and within the isolated species, reference sequences were selected based on Basic Local Alignment Search Tool (BLAST Version 2.16.0) results from the NCBI database and subsequently included in the analyses. In total, the final sequence alignment of the combined dataset included sequences from 66 *Alternaria* strains obtained in this study, along with 23 sequences of *Alternaria* reference strains retrieved from NCBI-BLAST. Additionally, to further ascertain the identity of the strains, a phylogenetic analysis was performed on the combined sequences of the two fragments. The combined dataset resulted in a total alignment of 871 sites comprising the *gpd* and *alt-a1* sequences.

**Figure 4:**
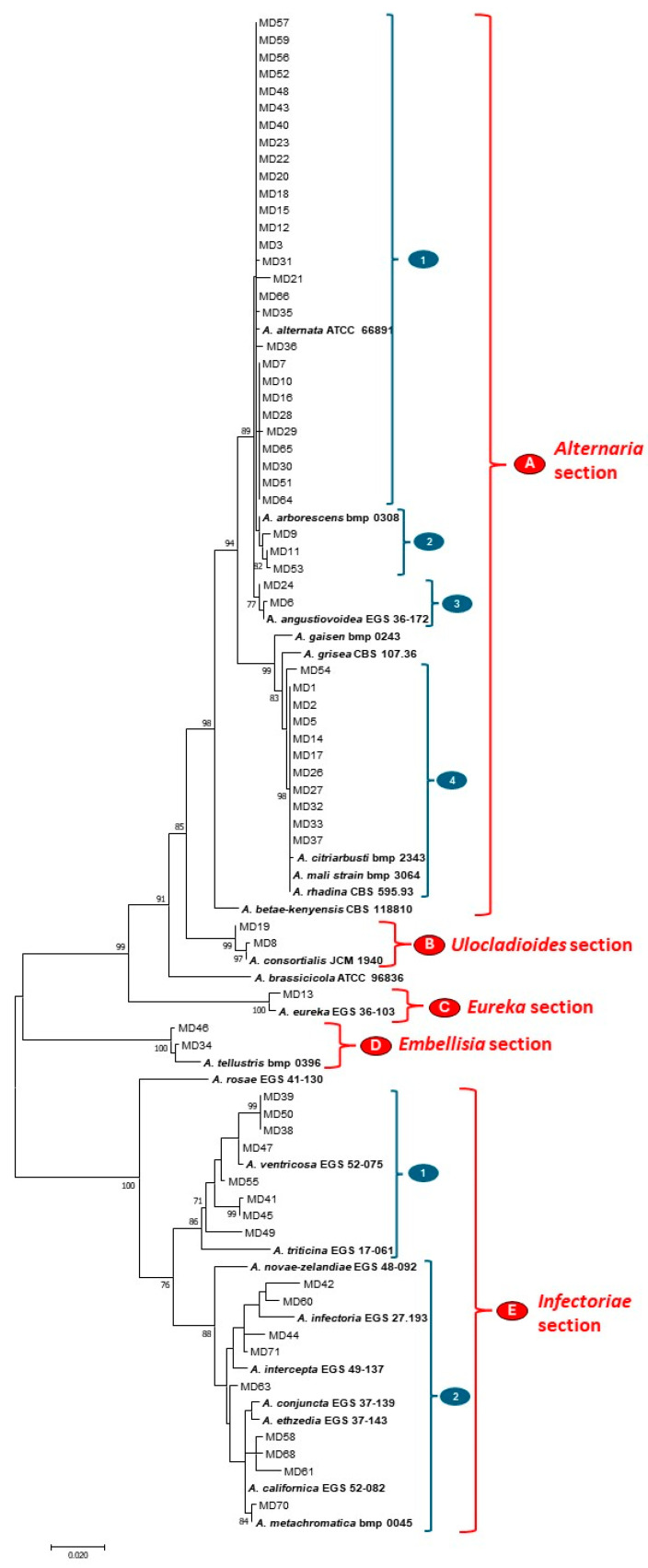
Phylogenetic tree generated by the maximum parsimony method (bootstrap 1000 replicates) of combined allergen Alt a1 and glyceraldehyde-3-phosphate dehydrogenase genes from 66 *Alternaria* strains isolated from Algerian wheat. Bootstrap values > 70 are shown next to the branches. Twenty-three *Alternaria* reference sequences were included in the analysis. Sub-clades and clades representing sections are also shown.

The resulting phylogenetic tree enabled the identification of five distinct clades, named from A to E in Figure 4, based on reference strains. These clades were supported by high bootstrap values (>85%). Moreover, each clade was clearly separated and corresponded to taxonomic sections, including *Alternaria*, *Ulocladioides*, *Eureka*, *Embellisia* and *Infectoriae* sections (Figure 4).

Forty-four out of sixty-six *Alternaria* strains (67%) clustered in section *Alternaria* and they are grouped into four well-supported sub-clades. Twenty-eight strains grouped with reference strain *A. alternata* ATCC 66891 in the sub-clade A1. A low variability was observed in this sub-clade, limited to the strains MD7, MD10, MD16, MD28, MD29, MD65, MD30, MD51 and MD64, that showed significant similarities among them but slightly diverged from the reference strain of *A. alternata*.

Three field strains were grouped with reference strain *A. arborescens* BMP 0308 in the sub-clade A2, and two field strains were grouped with reference strain *A. angustiovoidea* EGS 36-172 in the sub-clade A3. No strain clustered with the reference strains *A. gaisen* BMP 0243, *A. grisea* CBS 107.36 and *A. betae-kenyensis* CBS 118810. Sub-clade A4 included 11 field strains and the reference strains *A. citriarbusti* BMP 2343, *A. mali* BMP 3064 and *A. rhadina* CBS 595.93, but it was not possible to discriminate between the different morpho-species. Therefore, the isolates grouped within this cluster were not identified at the species level.

On the other hand, all strains clustering within *Ulocladioides*, *Eureka* and *Embellisia* sections (clades B, C and D, respectively) were well discriminated against and supported by high bootstrap values. In particular, two field strains, MD19 and MD8, grouped with the reference strain *A. consortialis* JCM 1940; the field strain MD13 clustered with *A. eureka* EGS 36-103, and the two field strains MD46 and MD34 showed high homology with *A. tellustris* BMP 0396. Reference strains representative of the sections *Brassicicola* (*A. brassicicola* ATCC 96836) and *Pseudoalternaria* (*A. rosae* EGS 41-130) included in the analysis did not cluster with any field strain.

Seventeen out of sixty-six *Alternaria* field strains (about 26%) were included in the *Infectoriae* section (clade E, Figure 4). However, most of the isolates showed very high variability either among themselves or with reference strains, grouped into two evident sub-clades. Sub-clade E1 included eight field strains, four strains of which (MD39, MD50, MD38 and MD47) were more closely related to the reference strain *A. ventricosa* EGS 52-075 (Figure 4). The sub-clade E2 included nine field strains and six reference strains, namely *A. infectoria* EGS 27.193, *A. intercepta* EGS 49-137, *A. conjuncta* EGS 37-139, *A. ethzedia* EGS 37-143, *A. californica* EGS 52-082 and *A. metachromatica* bmp 0045. Except for MD42 and MD60, which showed high homology with *A. infectoria,* and for the strain MD70, which was clearly identified as *A. metachromatica* (87% bootstrap value), this clade was characterized by very high variability among the strains and weak support of internal branches, causing difficulties in establishing the phylogenetic relationship between the Algerian strains and the species reference strains.

The six strains not included in the phylogenetic analysis, MD4, MD25, MD62, MD67, MD69 and MD72, were identified based on BLAST analysis. Moreover, a phylogenetic tree based on the single *gpd* gene was obtained and shown in Figure S1. All these strains were identified as belonging to the *Infectoriae* section, as reported in Table S2.

### 2.3. Mycotoxin Production of Alternaria Strains

All tested *Alternaria* strains, with the exception of the strains belonging to *Ulocladioides*, *Embellisia* and *Infectoriae* sections (Table S2), were able to synthesize at least one of the targeted mycotoxins AME, AOH, TeA, ATX-I, TEN and ALT. The production levels of AME and AOH varied significantly among the strains, ranging from 2 to 1019 mg kg^−1^ for AME and from 9 to 15950 mg kg^−1^ for AOH. Moreover, these mycotoxins co-occurred in 81% of the strains known as producers of either AME or AOH.

As reported in Table 2, the majority of the strains (70–100%) within the A1 and A2 phylogenetic sub-clades produced AME and AOH at high levels. In particular, strains from the A1 sub-clade had an average production of 211 mg kg^−1^ for AME and 980 mg kg^−1^ for AOH, while those in the A2 sub-clade produced 449 mg kg^−1^ and 574 mg kg^−1^ of AME and AOH, respectively. On the contrary, all strains belonging to the A3 and A4 sub-clades produced these mycotoxins at significantly lower levels: 109 and 34 mg kg^−1^, respectively, for A3, and 272 and 203 mg kg^−1^, respectively, for A4. No AME or AOH production was reported for the strains belonging to the other *Alternaria* sections.

Regarding TeA production, almost all the strains of the *Alternaria* section (clade A) showed a significant ability to produce this toxin, with levels ranging from 16 mg kg^−1^ (MD10) to 13165 mg kg^−1^ (MD26). The highest production of TeA was detected from the strains grouped within the sub-clade A4 (*A. citriarbusti*/*A. mali*/*A. rhadina*). All strains within the A4 sub-clade, except for a single strain (MD5), demonstrated significant capability to produce TeA, with levels up to 13165 mg kg^−1^ (mean value of 6794 mg kg^−1^). Also, the strains identified as *A. alternata* (sub-clade A1) were reported to produce a high amount of TeA, with values up to 9032 mg kg^−1^ (mean value of 2915 mg kg^−1^); however, five out of twenty-eight strains of *A. alternata* did not produce this mycotoxin. Two out of three *A. arborescens* strains produced TeA, with values ranging between 413 and 670 mg kg^−1^ (mean value of 361 mg kg^−1^), while the two *A. angustiovoidea* strains (sub-clade A3) produced 507 and 1100 mg kg^−1^ of TeA, respectively (Table 2). Among the strains included in the *Infectoriae* section (clade E), only one strain (MD70) produced TeA at a low level (291 mg kg^−1^).

ATX-I was detected only in 25 strains out of the 65 analyzed, with values ranging from 1 to 114 mg kg^−1^. Within the *Alternaria* section, *A. arborescens* strains did not produce ATX-I, while the highest mean value was detected for *A. angustiovoidea* strains (sub-clade A4).

The production of TEN was rarely detected; only nine strains included in the *Alternaria* and *Infectoriae* sections were able to produce it, with values ranging from 8 to 136 mg kg^−1^.

The production of ALT was reported in about 35% of the strains, showing variable values for species of the *Alternaria* section, while low values (1 and 15 mg kg^−1^) were detected for the single strain of *A. eureka* (MD13) and for a single producer strain of the *Infectoriae* section (MD44), respectively.

The single strain of *A. eureka* (clade C) produced very low levels of ATX-I (5 mg kg^−1^) and ALT (1 mg kg^−1^), while the strains belonging to clades B and E did not produce any mycotoxins. On the other hand, within clade E, which represents the *Infectoriae* section, only four strains produced mycotoxins, although at very low levels, as previously reported. In particular, the strains MD60, MD44 and MD63 produced ATX-I (21 mg kg^−1^), TEN (135 mg kg^−1^) and ALT (15 mg kg^−1^), respectively. Finally, the strain MD70, identified as *A. metachromatica*, showed co-production of TeA, ATX-I and TEN (291, 7 and 12 mg kg^−1^, respectively).

### 2.4. Content of Alternaria Mycotoxins in Grain Samples

The majority of grain samples were contaminated with a combination of the five mycotoxins AME, AOH, TeA, ATX-I and TEN, while ALT was not detected in any of the analyzed grain samples (Table S1).

In particular, AME and AOH were detected in 75 and 69% of grain samples, respectively, with values ranging between 50 and 980 µg kg^−1^ and between 25 and 425 µg kg^−1^, respectively. TeA was detected in 35% of the grain samples, ranging from 381 to 705 µg kg^−1^, while ATX-I was found only in four samples (8%), with values up to 1758 µg kg^−1^. Contamination by TEN was detected in 21% of the grain samples, with a mean value of 38 µg kg^−1^ (Table 3).

The co-occurrence of AME and AOH was reported with high contents in all the examined Algerian regions. AME was detected with the highest mean value (415 µg kg^−1^) in the Khenchela region (Table 4), while AOH was detected in the M’Sila region (203 µg kg^−1^). ATX-I and TEN toxins were only found in the Sètif and Biskra regions, with mean values of 71 and 57 µg kg^−1^, respectively, in Sètif, and 8 and 31 µg kg^−1^, respectively, in Biskra. The presence of TeA was reported in four regions, with values of 489, 210, 261 and 120 µg kg^−1^ in Bejaia, Sètif, Batna and Biskra, respectively (Table 4).

## 3. Discussion

*Alternaria* is one of the most prevalent fungal genera infecting wheat worldwide [4,13,27,28,29]. The high percentage of *Alternaria* contamination, compared to other fungal genera, such as *Fusarium* and *Cladosporium*, reported in the present study, confirms this finding. Moreover, the opposite occurrence between *Alternaria* and *Fusarium* in wheat kernels is also assessed, suggesting competitive interactions between the two fungal genera, as observed in previous investigations [4,30]. The high frequency of *Alternaria* species on wheat in Algeria was reported for the first time by Mokhtar and Dehimat [24] on three samples of local wheat seed. Even then, the importance of assessing the potential risk of *Alternaria* toxin exposure in wheat intended for human consumption has been highlighted. The levels of contamination of Algerian wheat detected in this study suggest paying more attention to this fungal genus. In each region, the percentage of contamination of wheat kernels accounted for mean values from 30 to 60%, with 40% of the samples showing contamination above 50%. According to phylogenetic analysis, most of the strains identified in this study belonged to the *Alternaria* (51%) and *Infectoriae* (40%) sections. Similar results were reported by Ramires et al. [9] and Masiello et al. [4], who identified species belonging to the *Alternata* and *Infectoriae* sections as the most frequently occurring on wheat. Within the *Alternaria* genus, *A. alternata* is the most widely distributed species. It is a plant pathogen that causes leaf spot disease in more than 100 distinct host plant species, as well as an important saprophyte on decaying organic matter [15]. The use of *gpd* and *alt-a1* in the multi-locus analyses enabled us to distinguish between various species within the Alternaria section. The predominant group, sub-clade 1, comprised twenty-eight field strains: nineteen identified as *A. alternata*, nine strains grouped closely with *A. alternata* but distinguishable, three strains identified as *A. arborescens* and two strains as *A. angustiovoidea*. Many previous studies support the findings of this study, as it is possible to distinguish well between *A. alternata*, *A*. *arborescens* and *A. angustiovoidea* species in the *Alternaria* section, whereas often there is no differentiation between *A. alternata*, *A. tenuissima*, *A. turkisafria* and *A. limoniasperae* even when using multiple genes such as *alt-a1*, *gpd*, translation elongation factor (*tef*) and β-tubulin (*tub*) [4,7,31]. Therefore, as proposed by Woudenberg et al. [15] after whole-genome and transcriptome analyses, the two taxa *A. alternata* and *A. tenuissima* can be merged into a single species, *A. alternata*, a conclusion later confirmed by Somma et al. [7]. In our study, a well-supported cluster within the *Alternaria* section is the sub-clade A4, which included 10 field strains and three reference strains of *A. mali*, *A. citriarbusti* and *A. rhadina*, but no distinction among species of this clade was observed. Similarly, in previous studies, no distinction was observed between different species of this clade by using each of the genes *alt-a1*, *gpd* and *tef* [4,9]. On the other hand, the distinction between different species within the clade of *A. mali* is almost clear when combined genes *alt-a1*, *gpd*, *tef*, *calmodulin* and *tub* are used [7,8]. Anyway, *A. mali* is clearly distinguished from *A. alternata* both in this study and in various other investigations mentioned above, although Woudenberg et al. [15] has proposed the synonymization of these two species. Therefore, several disagreements about the taxonomy of the *Alternaria* genus, according to molecular phylogeny, exist. The process of selecting suitable molecular markers for phylogeny and systematic analysis is complicated by evolutionary discrepancies, including lineage sorting and recombination, resulting in inconsistent outcomes for each locus studied [32]. These mismatches require the investigation of alternative and more accurate techniques for classifying the many species that are included in the *Alternaria* genus sections and the development of more phylogenetically informative molecular markers. Recently, Dettman et al. [33] selected specific genetic markers (ASA-10 and ASA-19) able to consistently classify the strains within the *Alternaria* section to species or lineages, which led to the supposition of recently occurring inter-lineage hybridization/recombination events, thus providing new insights into *Alternaria* species diversity and distribution.

Strains within the clades of the *Infectoriae* section show high heterogeneity and lower levels of similarity among themselves compared to those of other sections, especially *the Alternaria* section, in accordance with Lawrence et al. [34] and Somma et al. [7]. The *Infectoriae* section grouped seventeen field strains into two not-well-supported subclades but characterized by high variability. The strains were not recognized at the species level, with the exception of strain MD70, which was identified as *A. metachromatica*. Moreover, additional six field strains belong to this section, but they were not included in phylogenetic analysis since they failed in *alt-a1* gene amplification. Similar results occurred in a previous study [31], strengthening the hypothesis that polymorphisms within the annealing site of the primers could be the cause of failure. To accurately identify the species of the Algerian strains belonging to this section, additional genes should be sequenced to achieve improved results. However, since the species included in the *Infectoriae* section are not able to produce mycotoxins, and therefore the mycotoxigenic risk is absent and their pathogenicity ability is similar, their identification at the species level has not been considered important in this study. However, these species are responsible for about 40% of the contamination detected in the Algerian wheat kernels.

Among Algerian field strains, only six strains belonged to sections other than *Alternaria* and *Infectoriae*. Two strains were identified as *A. consortialis* in the *Ulocladioides* section, one as *A. eureka* in the *Eureka* section and two as *A. tellustris* in the *Embellisia* section. Previous research indicates that species from the *Ulocladioides*, *Eureka* and *Embellisia* sections are rarely isolated. For instance, isolates from the *Ulocladioides* section were found in date palms in southern Tunisia [17] and in Lebanese durum wheat grains [31]. Some strains belonging to the *Eureka* section were also isolated from *Solanaceae* plants in Algeria [35]. However, these species, namely *A. consortialis*, *A. eureka* and *A. tellustris*, were isolated for the first time in durum wheat in Algeria.

Based on species identification, the capability of the Algerian strains to produce mycotoxins reflects the shared knowledge, since most of the *A. alternata* strains produce high levels of AME, AOH and TeA [36]. Moreover, most of them (55%) produced moderate levels of ATX-I, TEN and ALT under in vitro conditions. On the contrary, all *A. consortialis* strains did not produce any mycotoxin, as previously reported by Rabaaoui et al. [17]. Similarly, *A. tellustris* and *A. eureka* were not able to synthesize these mycotoxins. Recently, Di Martino et al. [37] have demonstrated the activity of *A. tellustris* against two fungal pathogens of wheat root, *Fusarium oxysporum* and *Rhyzoctonia solani*. Moreover, *A. consortialis*, included in the *Ulocladioides* Section, and *A. embellisia*, phylogenetically very closely related to *A. tellustris*, have been reported to be able to improve maize seed germination and root length on maize seedlings [38]. In particular, *A. consortialis* produced a high level of indole-3-acetic acid-like compounds, reported as involved in plant growth-promoting [39]. Interestingly, *A. consortialis* isolated in this study did not produce detectable mycotoxins, supporting its potential as a non-toxigenic fungal endophyte. This opens the possibility for future exploration of their plant growth-promoting properties, provided that their genetic stability and biosafety are thoroughly assessed. Thus, the occurrence of these *Alternaria* species on wheat suggests the beneficial potential use of these species to control mycotoxigenic fungi and to promote the growth of cereal plants.

The chemical analysis on Algerian wheat allowed for the evaluation of the *Alternaria* mycotoxin occurrence and the subsequent real risk for Algerian consumers. A slight correlation could be observed based on the geographical origin of the wheat samples. In particular, ATX-I and TEN are absent in Batna, M’Sila and Khenchela regions, characterized by arid and cold climate conditions. Effectively, these mycotoxins are absent in the Bejaia region too, but a single sample was collected from this region, making it incorrect to derive an assumption related to the climate. Nevertheless, Algeria shares the same climatic conditions as other wheat-growing countries, such as Tunisia, Morocco, Egypt, Libya, Sudan and Saudi Arabia, countries poorly investigated for *Alternaria* species and their mycotoxin contamination. Thus, findings collected in other Mediterranean and semi-arid areas could be useful to predict *Alternaria* occurrence and distribution and for developing disease management strategies [40]. Furthermore, based on wheat variety, Simeto and Vitron showed higher contamination compared to Oued El Bared and Boussalem, although specific investigations should be planned with a higher number of samples if the effect of varietal choice has to be accurately assessed. However, it should be considered that Simeto was grown exclusively in the Batna region, and Vitron was the prevalent variety grown in M’Sila, Khenchela and Biskra, all regions characterized by arid climate. Thus, the differences detected among varieties could also depend on the geographical origin of the samples.

However, most of the wheat samples were contaminated with at least one *Alternaria* mycotoxin (85%), with the highest levels detected for AME and TeA. In addition, AME is the predominant mycotoxin, detected in 75% of the wheat samples, in agreement with previous studies in Argentina [28], China [41] and Egypt [40]. The levels of AME detected in Algerian samples were very variable, with a mean value higher than 300 µg kg^−1^, up to 980 µg kg^−1^. In previous studies on wheat, AME has been detected at levels ranging from 2 in Israel to 39 in Serbia [42], up to ten times less than our results. On the other hand, TeA has been detected at very high levels (up to 705 µg kg^−1^) in Algerian wheat, with a mean value of 177 µg kg^−1^, similar to previous reports in Germany [43] and in China [44].

Although AME and AOH are the most frequently detected *Alternaria* mycotoxins in wheat, ALT and TeA have high acute toxicity in vitro and in animal experiments [45]. In addition, other studies showed that TeA was responsible for the outbreak of a human hematologic disorder (onyalay) in Africa and esophageal cancer in China [46]. AME and AOH are less acutely toxic; however, they have been described to induce mutagenic, carcinogenic, cytotoxic and genotoxic effects. On the contrary, low levels of ATX-I and TEN with low frequency, and the absence of ALT have been detected in the Algerian wheat samples. ATX-I, although considered a minor *Alternaria* metabolite, has been reported to possess mutagenic properties [45], while acute toxicity related to ALT has been reported, further highlighting the health risks posed by *Alternaria* mycotoxins to humans [47]. However, only a few studies included these mycotoxins in monitoring the toxigenic risk on cereal commodities, detected at concentrations below 50 µg kg^−1^ [45,48,49]. Moreover, the absence of ALT contamination in wheat kernels was confirmed in previous studies [45,49]. If we consider the EU recommendation of 500 µg kg^−1^ of TeA and 2 µg kg^−1^ of AME and AOH for cereal-based food for infants, the data reported in the present study on Algerian wheat raise the urgent need for regulation including limits for *Alternaria* toxins in food and feed intended for human and animal consumption.

Furthermore, the co-occurrence of multiple toxins has been detected, in agreement with several investigations [50]. Many studies have reported additive or even synergic effects due to co-exposure to *Alternaria* toxins, demonstrating more pronounced oxidative damage induced by the combination of AOH and AME than the effect of individual toxins [51]. AME and AOH have been detected in 73% of the Algerian samples, while 27% showed the presence of both AME and TeA, and only 6% showed the combination TEN + TeA. In contrast, Xu et al. [52] reported that the combination of TEN + TeA, AME + TeA and AME + AOH accounted for 97%, 91% and 6% of the wheat flour samples, respectively, in China. In a 10-year survey in Germany, the co-occurrence of multiple *Alternaria* mycotoxins was found only in a few wheat samples, with TeA and AOH being the most frequent combination, while co-occurrence of AOH and AME was not detected [43]. The differences in frequency and distribution of *Alternaria* mycotoxins in wheat, detected in samples from different geographical areas of the world, highlight the important role played by climatic conditions and agronomic practices. In addition, two samples out of forty-eight showed simultaneous contamination with five mycotoxins—AME, AOH, TeA, ATX-I and TEN—and another two samples with four mycotoxins, while the co-occurrence of three *Alternaria* mycotoxins has been detected in 25% of the wheat samples. These results are of great concern, if we consider the possibility of additive/synergistic effects derived from co-contamination of multiple mycotoxins in food and feed material.

## 4. Conclusions

The phylogenetic analysis shows once again the complexity of the taxonomic recognition of the species belonging to the *Alternaria* genus. Nevertheless, this study highlights the great variability of the *Alternaria* species that contaminate Algerian durum wheat and thus the need to better investigate the pathogen population structure in order to develop effective strategies to reduce fungal and mycotoxin contamination of wheat in Algeria. Notably, *A. eureka*, *A. consortialis* and *A. tellustris* species have been detected for the first time in wheat in Algeria, with the latter two species potentially applicable for development in pest management.

Moreover, a considerable mycotoxigenic risk has been detected in Algeria, although some regions seem to be more contaminated than others by AME and AOH. In conclusion, the simultaneous detection of different *Alternaria* mycotoxins, together with the high frequency of detection and high values of the single mycotoxins in the wheat samples, highlights the urgent need for regulation of limits in food and feed. We recommend improving harvest and storage practices, monitoring environmental conditions, and implementing routine screening for *Alternaria* mycotoxins in wheat. These measures could help reduce contamination risks and improve food safety in Algeria.

## 5. Materials and Methods

### 5.1. Wheat Sampling and Isolation of Alternaria Strains

**Figure 5:**
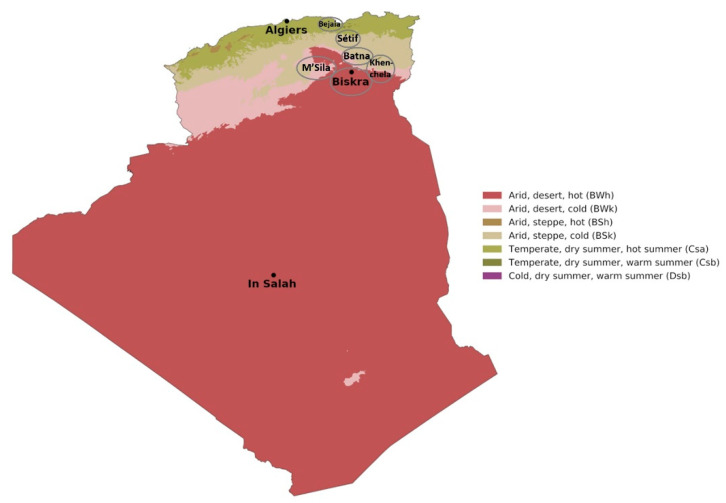
Map of Algerian regions in which sampling wheat fields were located, showing climate areas according to the Köppen–Geiger climate classification (Source: Beck et al. [53]).

During the 2021–2022 crop season, 48 durum wheat kernel samples (Table 1) were obtained from the National Center of Control and Certification (CNCC), regional laboratory of Sètif, Algeria. Samples belonged to six different durum wheat cultivars and were collected from wheat fields located in 6 regions of the northeast to the southeast of Algeria, characterized by different climatic conditions according to the Koppen–Geiger climate classification [53], as reported in Figure 5. Specifically, one single sample was collected from the Bejaia region, characterized by a temperate warm climate; 28 samples from Sètif, characterized by a transitional Mediterranean climate, with dry and hot summers; 5 samples were collected in the Batna region, whose climate is classified as arid, steppe and cold; 5 and 2 samples were collected in M’Sila and Khenchela regions, respectively, both classified as an arid, desert, cold climate; and 7 samples were collected from the Biskra region, characterized by an arid, desert, hot climate (Table 1).

For each sample, 1 kg of kernels was randomly collected from the storage center by mixing the batch derived from each field. For fungal isolation, a sub-sample of kernels was surface disinfected by immersion in 2% sodium hypochlorite solution for 2 min, followed by two rinses with sterile distilled water and then dried on sterile filter paper. One hundred kernels of each wheat sample were transferred into Petri dishes containing potato dextrose agar (PDA) medium amended with streptomycin sulphate salt (100 mg L^−1^) and neomycin (50 mg L^−1^). Fungal colonies developed from kernels after incubation at 25 °C for seven days were morphologically identified at genus level. The fungal contamination by each fungal genus was calculated as the percentage of kernels contaminated out of the total number of plated kernels. The colonies identified as *Alternaria* were single-spore purified and grown on PDA at 25 °C for further analyses.

### 5.2. Molecular Characterization of Alternaria Strains

Seventy-two *Alternaria* strains were selected, among 608 isolated strains, for molecular characterization. The mycelium of three-day-old fungal colonies, grown on sterile cellophane disks overlaid on PDA, was collected by scraping and lyophilized.

Genomic DNA was extracted and purified from powdered lyophilized mycelia (10–15 mg) by using the “Wizard Magnetic DNA Purification System for Food” kit (Promega Corporation, Madison, WI, USA), according to the manufacturer’s protocol. Quantity and integrity of DNA were checked at Thermo-Scientific Nanodrop (LabX, Midland, ON, Canada) and by comparison with a standard DNA (1 kb DNA Ladder, Fermentas GmbH, St. Leon-Rot, Germany) on 0.8% agarose gel after electrophoretic separation.

The two most informative genes, Allergen Alt-a1 (*alt-a1*) and glyceraldehyde 3-phosphate dehydrogenase (*gpd*), were selected for the molecular characterization and for studying a reliable phylogenetic relationship among *Alternaria* strains using a multi-locus sequence approach.

For each strain, the two genes *alt-a1* and *gpd* were amplified by using the primer pairs Alt-for/Alt-rev [54] and gpd1/gpd2 [55], respectively. PCR reactions were carried out in a final volume of 15 μL, containing 0.125 μL of Start Taq DNA Polymerase (1 U/μL; Fisher Molecular Biology, Rome, Italy), 0.45 μL of each primer (10 μM), 1.2 μL of 10 mM dNTPs, 1.5 μL of 10X buffer and 30 ng of fungal genomic DNA, using the PCR conditions reported by Masiello et al. [4].

PCR products were enzymatically purified through a mixture of EXO/FastAP enzymes (Exonuclease I, FastAP thermosensitive alkaline phosphatase; ThermoFisher Scientific, Waltham, Massachusetts, USA), according to the manufacturer’s recommendations. Subsequently, each PCR product was labeled using the Big Dye Terminator Cycle Sequencing Ready Reaction Kit (Applied Biosystems, Foster City, CA, USA), according to the manufacturer’s protocol. After purification by filtration through 5% Sephadex G-50 (Sigma-Aldrich, Milan, Italy) columns, each strand was sequenced in an ABI PRISM 3730 Genetic Analyzer (Applied Biosystems, Foster City, CA, USA).

DNA sequences were analysed with the Sequencing Analysis 5.2 software (Applied Biosystems, Waltham, MA, USA). Each consensus sequence was generated from the forward and reverse strands with Bionumerics software (Applied Maths, Kortrijk, Belgium). Initially, FASTA sequences were compared with the available sequences in the National Center for Biotechnology Information (NCBI) database using the Basic Local Alignment Search Tool (BLAST). Then, phylogenetic analysis was conducted with MEGA7 software, using the Maximum Likelihood method and bootstrap analysis based on a heuristic search with 1000 replicates and gap removal [56]. To resolve phylogenetic relationships among and within detected species, reference sequences properly selected based on BLAST search were downloaded from the NCBI database and included in the analyses.

### 5.3. Mycotoxin Detection in Alternaria Culture Materials and Wheat Samples

The method described by Solfrizzo et al. [57] for the analysis of *Alternaria* toxins in carrots, with slight modifications proved to be suitable for the analysis of AME, AOH, TeA, ATX-I, TEN and ALT in the *Alternaria* rice cultures as well as in wheat samples. Chemical analysis for 65 *Alternaria* strains, molecularly identified and characterized, was carried out to evaluate their capability to produce, under experimental conditions, AME, AOH, TeA, ATX-I, TEN and ALT [58]. Flasks containing 30 g of rice, hydrated to 45% moisture with distilled water, were autoclaved at 121 °C for 30 min. For each *Alternaria* strain, five mycelium plugs (4 mm), collected from the margin of actively growing colonies, were used to inoculate the rice and incubated in darkness at 25 °C for three weeks. Then, the rice cultures were dried at 65° for two days. The samples were finely ground with an Oster Classic grinder (220–240 V, 50/60 Hz, 600 W; Madrid, Spain). In the case of culture samples, two grams of homogenized samples were extracted with 10 mL of acetonitrile/methanol/water mixture (45:10:45, v/v/v), adjusted to pH 3 with hydrochloric acid. The tubes were shaken for 60 min with an orbital shaker and centrifuged at 4500 rpm for 10 min. After diluting the extract ten times with distilled water, it was further centrifuged at 15,000 rpm for 15 min at 4 °C before analysis by reversed-phase HPLC with a UV/DAD detector.

With regard to the 48 wheat samples collected from Algerian regions, five grams of finely ground grains were added to 30 mL of extraction solvent mixture (acetonitrile/methanol/water) and shaken for 60 min. Then, the supernatant was filtered through Whatman No. 4 filter paper (Whatman, Maidstone, UK) and collected for the clean-up step.

#### 5.3.1. Solid Phase Extraction (SPE) Clean-Up for TeA

The SPE clean-up of TeA was carried out on an Oasis^®^ HLB column (Waters Corporation, Milford, CT, USA). A volume of 5 mL of the filtrate was added to 10 mL of sodium dihydrogen phosphate solution and mixed, and an aliquot of 7.5 mL of the diluted filtrate was passed through the Oasis^®^ HLB column, previously conditioned with 2 mL acetonitrile, at a rate of approximately 1–2 drops per second. Then, TeA was eluted with 1 mL of the elution solvent (acetonitrile/methanol/0.05 M dihydrogen phosphate solution, 35:35:30, v/v/v), collected in a vial and stored at approximately 4 °C until HPLC analysis.

#### 5.3.2. Solid Phase Extraction (SPE) Clean-Up for AME, AOH, ATX-I, TEN and ALT

The SPE clean-up of AME, AOH, ATX-I, TEN and ALT was carried out on a C18 column (Waters Corporation). A volume of 2.5 mL of the filtrate was pipetted through the column, which had been previously conditioned with 6 mL of acetonitrile, and the eluate was discarded. Mycotoxins were eluted into a 4 mL vial positioned under the column with 2 mL of elution solvent (acetonitrile/glacial acetic acid, 99:1, v/v). The eluate was evaporated to dryness under a nitrogen stream at 60 °C. The purified residue was then dissolved in 500 µL of methanol by vortexing for 1 min, diluted with 500 µL of the HPLC mobile phase (Solvent A) and stored at approximately 4 °C until HPLC analysis.

#### 5.3.3. Recovery Experiments

Recovery experiments were performed in triplicate at a level of 1.0 µg/g by spiking a blank (*Alternaria* toxins-free) durum wheat sample with 100 µL of a stock solution containing 0.05 µg/µL TeA, AME, AOH, TEN and ALT in methanol. After spiking, samples were left for 30 min at room temperature to allow solvent evaporation, then separately extracted and cleaned up through SPE columns as described above.

The HPLC/DAD determination and confirmation of *Alternaria* toxins was performed according to the procedure described by Solfrizzo et al. [57]. Each extract (50 µL) was injected into the HPLC Agilent 1260 equipped with a binary pump and a column thermostat set at 30 °C. The analytical column was a Symmetry Shield RP18 150 × 4.6 mm, 5 µm (Waters). The mobile phase consisted of a binary gradient applied as follows: the initial composition of the mobile phase—100% of (A) acetonitrile/0.027 M sodium dihydrogen phosphate (25:75, v/v)/0% of (B) acetonitrile/0.027 M sodium dihydrogen phosphate (50:50, v/v)—was kept constant for 5 min, followed by solvent B at 100% from 5.1 to 25 min. The flow rate of the mobile phase was set at 1 mL/min. The UV diode array detector was set at wavelengths of 279 nm and 256 nm. The retention time for *Alternaria* toxins was about 7.8 min for ANE; 8.4 min for TeA, 8.6 min for TEN, 9.2 min for ATX-I, 12.5 min for AOH and 18 min for AME. The calibration curve showed good linearity in the range 0.01–1.00 µg/mL. The limit of quantification (LOQ) for wheat samples was 0.02 µg/g for ANE, TEN, ATX-I, AOH and AME and 0.1 µg/g for TeA. The LOQ for fungal strains was 0.5 µg/g for ANE, TEN, ATX-I, AOH and AME and 2.5 µg/g for TeA. Recovery was higher than 73%, with relative standard deviations less than 7%.

## Figures and Tables

**Table 1:** Geographical origin of Algerian durum wheat samples. The names of the regions, with relative climate conditions, the number of samples and the wheat varieties are also reported.

Region	Climate Conditions	No. of Total Samples	Wheat Variety	No. of Samples	Wheat Sample
Bejaia	Temperate, dry and warm summer	1	Boussalem	1	V30
Sètif	Temperate, dry and hot summer	28	Boussalem	11	V8, V10, V12, V14, V21, V23, V25, V26, V27, V36, V42
			GTA Dur	1	V9
			Mohamed Ben Bachir	1	V32
			Oued El Bared	15	V4, V15, V16, V17, V18, V19, V20, V22, V28, V31, V39, V43, V46, V47, V48
Batna	Arid, steppe, cold	5	Oued El Bared	2	V29, V35
			Simeto	3	V24, V33, V34
M’Sila	Arid, desert, cold	5	Vitron	5	V1, V2, V3, V7, V11
Khenchela	Arid, desert, cold	2	Vitron	2	V41, V44
Biskra	Arid, desert, hot	7	Oued El Bared	2	V38, V40
			Vitron	5	V5, V6, V13, V37, V45

**Table 2:** Alternariol (AOH), alternariol methyl ether (AME), tenuazonic acid (TeA), altertoxin I (ATX-I), tentoxin (TEN) and altenuene (ALT) production by *Alternaria* strains isolated from wheat in Algeria. Based on phylogenetic clades, the percentage of the producer strains, with the relative mean values and range of production expressed in mg kg^−1^, are reported.

	Phylogenetic Clade		AME	AOH	TeA	ATX-I	TEN	ALT
*Alternaria* section	Sub-clade A1 (27 strains)	Producer strains (%)	67	70	85	41	15	55
		Mean (mg kg^−1^)	211	980	2915	27	63	85
		Range (mg-kg^−1^)	18–564	9–15949	16–9032	1–87	8–136	1–395
	Sub-clade A2 (3 strains)	Producer strains (%)	100	67	67	0	0	100
		Mean (mg kg^−1^)	449	574	542	0	0	344
		Range (mg-kg^−1^)	192–878	423–724	413–670	0	0	212–423
	Sub-clade A3 (2 strains)	Producer strains (%)	100	100	100	100	50	100
		Mean (mg kg^−1^)	109	34	804	111	10	206
		Range (mg-kg^−1^)	17–200	21–48	507–1100	109–114	10	99–313
	Sub-clade A4 (11 strains)	Producer strains (%)	100	100	91	73	18	36
		Mean (mg kg^−1^)	272	203	6794	34	19	71
		Range (mg-kg^−1^)	2–1019	20–508	90–13,165	5–76	8–30	7–192
*Eureka* section	Clade C (1 strain)	Producer strains (%)	0	0	0	100	0	100
		Mean (mg kg^−1^)	0	0	0	5	0	1
		Range (mg-kg^−1^)	0	0	0	5	0	1
*Infectoriae* section	Clade E (17 strains)	Producer strains (%)	0	0	6	18	12	6
		Mean (mg kg^−1^)	0	0	291	11	74	15
		Range (mg-kg^−1^)	0	0	291	4–21	12–135	15

**Table 3:** Mycotoxin occurrence in 48 durum wheat samples from Algeria.

Mycotoxin	Contaminated Samples (%)	Mycotoxin Content (µg kg^−1^)	
		Mean Value	Range
AME	75	314	50–980
AOH	69	44	25–425
TeA	35	177	381–705
ATX-I	8	43	58–1758
TEN	21	38	52–321

AME = alternariol monomethyl ether; AOH = alternariol; TeA = tenuazonic acid; ATX-I = altertoxin I; TEN = tentoxin.

**Table 4:** Mycotoxin amount (µg kg^−1^) detected in durum wheat samples collected in six regions of Algeria. The percentage of contaminated samples out of the total analyzed samples, mean values and range of mycotoxin occurrence calculated on positive samples are shown for each Algerian region.

Region	No. of Samples		AME	AOH	TeA	ATX-I	TEN
Bejaia	1	Contaminated samples (%)	0	100	100	0	0
		Mean value (µg kg^−1^)	0	35	489	0	0
		Range (µg kg^−1^)	0	35	489	0	0
Sètif	28	Contaminated samples (%)	82	93	39	11	32
		Mean value (µg kg^−1^)	348	18	210	71	57
		Range (µg kg^−1^)	50–820	25–45	381–705	82–1758	52–321
Batna	5	Contaminated samples (%)	40	40	60	0	0
		Mean value (µg kg^−1^)	370	13	261	0	0
		Range (µg kg^−1^)	870–980	25–40	407–471	0	0
M’Sila	5	Contaminated samples (%)	60	60	0	0	0
		Mean value (µg kg^−1^)	224	203	0	0	0
		Range (µg kg^−1^)	205–365	275–425	0	0	0
Khenchela	2	Contaminated samples (%)	100	100	0	0	0
		Mean value (µg kg^−1^)	415	40	0	0	0
		Range (µg kg^−1^)	370–460	40	0	0	0
Biskra	7	Contaminated samples (%)	71	86	29	14	14
		Mean value (µg kg^−1^)	218	15	120	8	31
		Range (µg kg^−1^)	180–380	25	394–444	58	216

AME = alternariol monomethyl ether; AOH = alternariol; TeA = tenuazonic acid; ATX-I = altertoxin I; TEN = tentoxin.

## Data Availability

The original contributions presented in this study are included in the article/Supplementary Material. Further inquiries can be directed to the corresponding author.

## Supplementary Materials

The following supporting information can be downloaded at: https://www.mdpi.com/article/10.3390/toxins17060309/s1, Figure S1: Phylogenetic tree generated by the maximum parsimony method (bootstrap 1000 replicates) of glyceraldehyde-3-phosphate dehydrogenase gene sequences of 23 *Alternaria* Algerian strains belonging to the *Infectoriae* section, isolated from Algerian wheat. Bootstrap values > 70 are shown next to the branches. Seventeen *Alternaria* reference sequences from type material were included in the analysis. *Alternaria alternata* ATCC 11680 reference strain was used as an outgroup. Table S1: Mycotoxin amounts, expressed in mg kg^−1^, detected in 48 durum wheat samples collected from different regions of Algeria. The mycotoxins analyzed were alternariol monomethyl ether (AME), alternariol (AOH), tenuazonic acid (TeA), altertoxin I (ATX-I), tentoxin (TEN) and altenuene (ALT). ALT was never detected in any wheat sample. Table S2: List of *Alternaria* strains isolated from different varieties of durum wheat samples collected from different regions of Algeria. The phylogenetic clade obtained from *alt-a1* and *gpd* gene sequencing, and the capability to produce alternariol monomethyl ether (AME), alternariol (AOH), tenuazonic acid (TeA), altertoxin I (ATX-I), tentoxin (TEN) and altenuene (ALT) mycotoxins are also reported.
