# Supplementary material for: Assessing Alternaria Species and Related Mycotoxin Contamination in Wheat in Algeria: A Food Safety Risk
Source: Toxins (Basel). 2025 Jun 18;17(6):309. doi: 10.3390/toxins17060309 (PMC12197503; doi:10.3390/toxins17060309)
Supplement: Supplementary file 1 [file toxins-17-00309-s001.zip › Table S1.pdf]

Table S1. Mycotoxin amount, expressed in mg kg^{-1}, detected in 48 durum wheat samples collected in different regions of Algeria. The mycotoxins analysed were alternariol monomethyl ether (AME), alternariol (AOH), tenuazonic acid (TeA), altertoxin I (ATX-I), tentoxin (TEN) and altenuene (ALT). ALT has never been detected in any wheat sample.

Wheat variety	Region	Wheat sample	AME	AOH	TeA	ATX-I	TEN
Boussalem	Bejaia	V30	n.d.	1.06	0.49	n.d.	n.d.
Boussalem	Sètif	V12	n.d.	n.d.	n.d.	n.d.	n.d.
Boussalem	Sètif	V10	n.d.	0.41	n.d.	n.d.	n.d.
Boussalem	Sètif	V14	n.d.	0.42	n.d.	n.d.	0.10
Boussalem	Sètif	V8	8.94	0.41	n.d.	n.d.	0.32
Boussalem	Sètif	V42	12.31	0.45	n.d.	n.d.	n.d.
Boussalem	Sètif	V26	12.49	0.40	n.d.	n.d.	n.d.
Boussalem	Sètif	V25	16.27	0.49	n.d.	n.d.	n.d.
Boussalem	Sètif	V36	18.37	1.15	0.38	n.d.	n.d.
Boussalem	Sètif	V23	15.51	1.28	0.45	n.d.	n.d.
Boussalem	Sètif	V27	10.40	0.57	0.48	n.d.	n.d.
Boussalem	Sètif	V21	15.57	0.62	0.49	n.d.	0.05
GTA Dur	Sètif	V9	10.62	0.46	n.d.	n.d.	0.18
Mohamed Ben Bachir	Sètif	V32	18.92	0.40	n.d.	n.d.	n.d.
Oued El Bared	Sètif	V4	n.d.	n.d.	n.d.	n.d.	n.d.
Oued El Bared	Sètif	V28	0.36	0.52	n.d.	n.d.	n.d.
Oued El Bared	Sètif	V43	6.90	0.85	n.d.	n.d.	n.d.
Oued El Bared	Sètif	V15	9.77	0.63	n.d.	n.d.	0.16
Oued El Bared	Sètif	V16	14.36	0.53	n.d.	n.d.	n.d.
Oued El Bared	Sètif	V31	16.79	0.71	0.41	n.d.	n.d.
Oued El Bared	Sètif	V39	7.90	0.48	0.48	n.d.	n.d.
Oued El Bared	Sètif	V22	11.95	0.47	0.50	0.08	0.20
Oued El Bared	Sètif	V18	13.23	1.20	0.61	0.16	0.16
Oued El Bared	Sètif	V19	0.02	0.28	0.68	n.d.	n.d.
Oued El Bared	Sètif	V17	12.71	0.13	0.69	n.d.	n.d.
Oued El Bared	Sètif	V20	12.39	0.72	0.71	1.76	n.d.
Oued El Bared	Sètif	V46	n.d.	0.86	n.d.	n.d.	0.24
Oued El Bared	Sètif	V47	9.79	1.16	n.d.	n.d.	n.d.
Oued El Bared	Sètif	V48	14.51	0.65	n.d.	n.d.	0.20
Oued El Bared	Batna	V29	n.d.	n.d.	n.d.	n.d.	n.d.
Oued El Bared	Batna	V35	19.09	0.26	0.41	n.d.	n.d.
Simeto	Batna	V33	n.d.	n.d.	n.d.	n.d.	n.d.
Simeto	Batna	V34	n.d.	1.32	0.43	n.d.	n.d.
Simeto	Batna	V24	26.03	n.d.	0.47	n.d.	n.d.
Vitron	M'Sila	V7	n.d.	n.d.	n.d.	n.d.	n.d.
Vitron	M'Sila	V3	8.07	0.43	n.d.	n.d.	n.d.
Vitron	M'Sila	V11	11.03	0.28	n.d.	n.d.	n.d.
Vitron	M'Sila	V2	12.14	0.32	n.d.	n.d.	n.d.
Vitron	M'Sila	V1	n.d.	n.d.	n.d.	n.d.	n.d.
Vitron	Khenchela	V41	14.54	0.79	n.d.	n.d.	n.d.
Vitron	Khenchela	V44	11.36	0.41	n.d.	n.d.	n.d.

Oued El Bared	Biskra	V40	10.56	0.46	n.d.	n.d.	n.d.
Oued El Bared	Biskra	V38	11.57	0.38	n.d.	n.d.	n.d.
Vitron	Biskra	V13	n.d.	n.d.	n.d.	n.d.	n.d.
Vitron	Biskra	V5	9.72	0.69	n.d.	0.06	n.d.
Vitron	Biskra	V6	10.62	0.36	n.d.	n.d.	0.22
Vitron	Biskra	V37	n.d.	0.31	0.39	n.d.	n.d.
Vitron	Biskra	V45	7.65	0.38	0.44	n.d.	n.d.
