# Supplementary material for: Assessing Alternaria Species and Related Mycotoxin Contamination in Wheat in Algeria: A Food Safety Risk
Source: Toxins (Basel). 2025 Jun 18;17(6):309. doi: 10.3390/toxins17060309 (PMC12197503; doi:10.3390/toxins17060309)
Supplement: Supplementary file 1 [file toxins-17-00309-s001.zip › Table S2.pdf]

Table S2. List of *Alternaria* strains isolated from different varieties of durum wheat samples collected from different regions of Algeria. The phylogenetic clade obtained from *alt-a1* and *gpd* gene sequencing and the capability to produce alternariol monomethyl ether (AME), alternariol (AOH), tenuazonic acid (TeA), altertoxin I (ATX-I), tentoxin (TEN) and altenuene (ALT) mycotoxins are also reported.

Strain code	Wheat sample	Wheat variety	Region	Section	Phylogenetic clade	Produced mycotoxins
MD56	V30	Boussalem	Béjaia	*Alternaria*	A1	AME, ALT
MD26	V30	Boussalem	Béjaia	*Alternaria*	A4	AME, AOH, TeA, ATX-I
MD43	V42	Boussalem	Sétif	*Alternaria*	A1	AME, AOH, TeA
MD3	V36	Boussalem	Sétif	*Alternaria*	A1	AME, AOH, TeA, ATX-I, ALT
MD10	V27	Boussalem	Sétif	*Alternaria*	A1	AME, AOH, TeA, ATX-I, TEN, ALT
MD9	V21	Boussalem	Sétif	*Alternaria*	A2	AME, TeA, ALT
MD37	V12	Boussalem	Sétif	*Alternaria*	A4	AME, AOH, TeA
MD32	V12	Boussalem	Sétif	*Alternaria*	A4	AME, AOH, TeA, ALT
MD33	V14	Boussalem	Sétif	*Alternaria*	A4	AME, AOH, TeA, ALT
MD14	V25	Boussalem	Sétif	*Alternaria*	A4	AME, AOH, TeA, ATX-I, ALT
MD44	V26	Boussalem	Sétif	*Infectoriae*	E	ALT
MD63	V10	Boussalem	Sétif	*Infectoriae*	E	ATX-I, TEN
MD45	V12	Boussalem	Sétif	*Infectoriae*	E	n.d.
MD50	V23	Boussalem	Sétif	*Infectoriae*	E	n.d.
MD58	V12	Boussalem	Sétif	*Infectoriae*	E	n.d.
MD68	V26	Boussalem	Sétif	*Infectoriae*	E	n.d.
MD71	V14	Boussalem	Sétif	*Infectoriae*	E	n.d.
MD25	V27	Boussalem	Sétif	*Infectoriae*	-	n.d.
MD62	V12	Boussalem	Sétif	*Infectoriae*	-	-
MD70	V12	Boussalem	Sétif	*Infectoriae*	E	TeA, ATX-I, TEN
MD36	V9	Gta Dur	Sétif	*Alternaria*	A1	TeA
MD46	V9	Gta Dur	Sétif	*Embellisia*	D	n.d.
MD69	V9	Gta Dur	Sétif	*Infectoriae*	-	ALT
MD72	V9	Gta Dur	Sétif	*Infectoriae*	-	n.d.
MD12	V46	Oued El Bared	Sétif	*Alternaria*	A1	ALT
MD57	V31	Oued El Bared	Sétif	*Alternaria*	A1	AME, AOH, TeA, ALT
MD16	V17	Oued El Bared	Sétif	*Alternaria*	A1	AME, AOH, TeA, ATX-I
MD35	V47	Oued El Bared	Sétif	*Alternaria*	A1	AME, AOH, TeA, ATX-I, ALT
MD7	V39	Oued El Bared	Sétif	*Alternaria*	A1	AME, AOH, TeA, ATX-I, ALT
MD29	V48	Oued El Bared	Sétif	*Alternaria*	A1	AOH, TeA, ALT
MD22	V28	Oued El Bared	Sétif	*Alternaria*	A1	AOH, TeA, ATX-I
MD24	V22	Oued El Bared	Sétif	*Alternaria*	A3	AME, AOH, TeA, ATX-I, ALT
MD6	V17	Oued El Bared	Sétif	*Alternaria*	A3	AME, AOH, TeA, ATX-I, TEN, ALT
MD17	V46	Oued El Bared	Sétif	*Alternaria*	A4	AME, AOH, TeA, ATX-I
MD2	V46	Oued El Bared	Sétif	*Alternaria*	A4	AME, AOH, TeA, ATX-I, TEN
MD19	V20	Oued El Bared	Sétif	*Ulocladioides*	B	n.d.
MD13	V39	Oued El Bared	Sétif	*Eureka*	C	ATX-I, ALT
MD34	V43	Oued El Bared	Sétif	*Embellisia*	D	n.d.
MD42	V22	Oued El Bared	Sétif	*Infectoriae*	E	n.d.

MD49	V47	Oued El Bared	Sétif	*Infectoriae*	E	n.d.
MD4	V16	Oued El Bared	Sétif	*Infectoriae*	-	n.d.
MD67	V18	Oued El Bared	Sétif	*Infectoriae*	-	n.d.
MD31	V29	Oued El Bared	Batna	*Alternaria*	A1	AME, AOH, TeA, ALT
MD5	V24	Simeto	Batna	*Alternaria*	A4	AME, AOH, ATX-I, ALT
MD60	V34	Simeto	Batna	*Infectoriae*	E	ATX-I
MD61	V33	Simeto	Batna	*Infectoriae*	E	n.d.
MD23	V38	Oued El Bared	Biskra	*Alternaria*	A1	AME, AOH, TeA, TEN, ALT
MD21	V38	Oued El Bared	Biskra	*Alternaria*	A1	TeA, ATX-I
MD48	V3	Vitron	M'Sila	*Alternaria*	A1	AME, AOH, TeA
MD66	V7	Vitron	M'Sila	*Alternaria*	A1	AME, AOH, TeA, ALT
MD20	V1	Vitron	M'Sila	*Alternaria*	A1	AME, AOH, TeA, ATX-I
MD64	V11	Vitron	M'Sila	*Alternaria*	A1	AME, ATX-I
MD40	V7	Vitron	M'Sila	*Alternaria*	A1	AME, TeA
MD15	V7	Vitron	M'Sila	*Alternaria*	A1	TeA
MD11	V2	Vitron	M'Sila	*Alternaria*	A2	AME, AOH, ALT
MD54	V7	Vitron	M'Sila	*Alternaria*	A4	AME, AOH, TeA, ATX-I
MD1	V1	Vitron	M'Sila	*Alternaria*	A4	AME, AOH, TeA, ATX-I, TEN
MD38	V3	Vitron	M'Sila	*Infectoriae*	E	n.d.
MD39	V3	Vitron	M'Sila	*Infectoriae*	E	n.d.
MD47	V7	Vitron	M'Sila	*Infectoriae*	E	n.d.
MD18	V44	Vitron	Khenchela	*Alternaria*	A1	AME, AOH, TeA, ATX-I, ALT
MD52	V44	Vitron	Khenchela	*Alternaria*	A1	AME, AOH, TeA
MD28	V44	Vitron	Khenchela	*Alternaria*	A1	ALT
MD27	V44	Vitron	Khenchela	*Alternaria*	A4	AME, AOH, TeA, ATX-I
MD55	V41	Vitron	Khenchela	*Infectoriae*	E	n.d.
MD59	V40	Oued El Bared	Biskra	*Alternaria*	A1	AOH, TeA, TEN, ALT
MD65	V6	Vitron	Biskra	*Alternaria*	A1	-
MD30	V37	Vitron	Biskra	*Alternaria*	A1	AME, AOH, TeA, ATX-I, TEN, ALT
MD51	V5	Vitron	Biskra	*Alternaria*	A1	AOH, TeA
MD53	V6	Vitron	Biskra	*Alternaria*	A2	AME, AOH, TeA, ALT
MD8	V5	Vitron	Biskra	*Ulocladioides*	B	n.d.
MD41	V5	Vitron	Biskra	*Infectoriae*	E	n.d.
