# Supplementary figures and images for: Assessing Alternaria Species and Related Mycotoxin Contamination in Wheat in Algeria: A Food Safety Risk
Source: Toxins (Basel). 2025 Jun 18;17(6):309. doi: 10.3390/toxins17060309 (PMC12197503; doi:10.3390/toxins17060309)

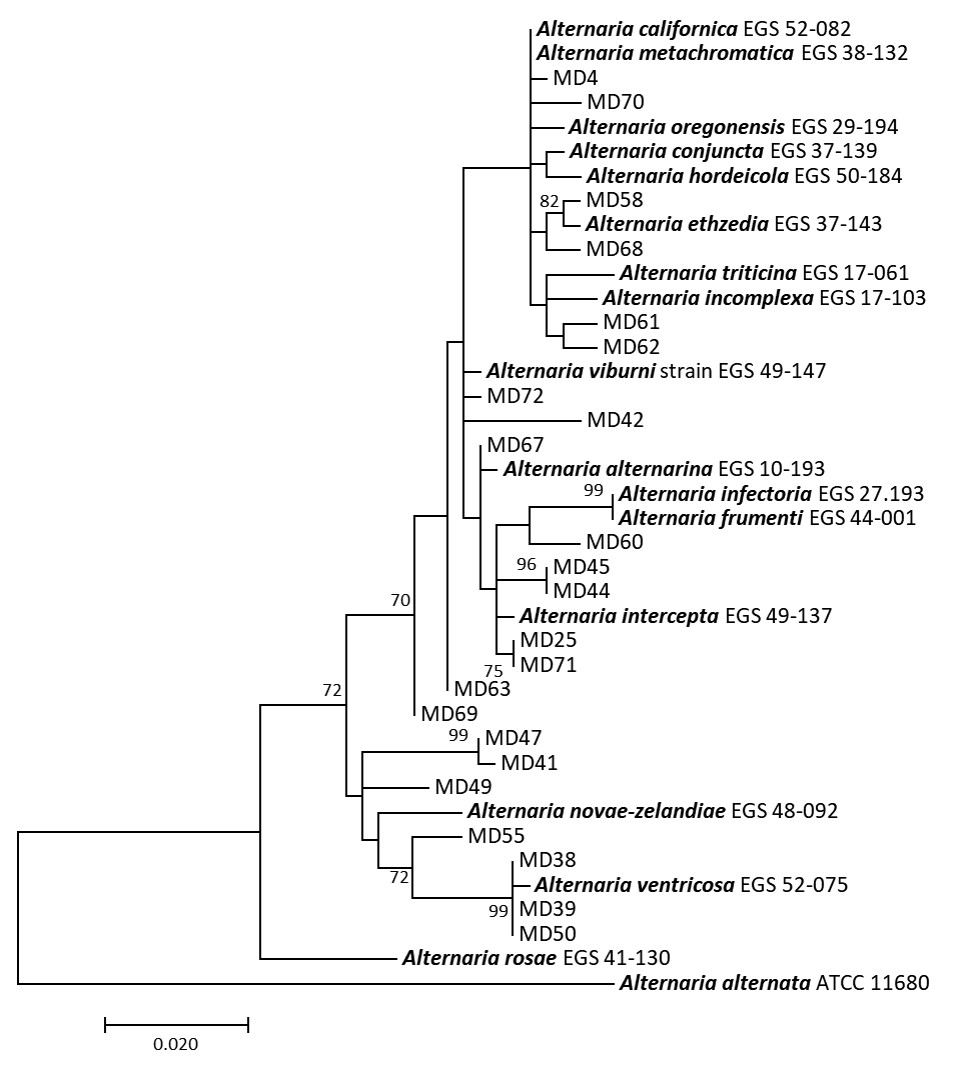

Supplement: Supplementary file 1 [file toxins-17-00309-s001.zip › Figure S1.jpg]
